# Temporally integrated multiomics analysis elucidates intricate regulatory mechanisms of ASFV in a wild boar lung-derived clonal cell line

**DOI:** 10.1186/s13567-025-01629-2

**Published:** 2025-10-14

**Authors:** Hua Wang, Miaomiao Ye, Wenlian Weng, Jiajun Wu, Yajin Qu, Peng Gao, Yongning Zhang, Lei Zhou, Xinna Ge, Xin Guo, Jun Han, Hanchun Yang

**Affiliations:** 1 State Key Laboratory of Veterinary Public Health and Safety, Key Laboratory of Animal Epidemiology of the Ministry of Agriculture and Rural Affairs, China Agricultural University College of Veterinary Medicine, Beijing, 100193 China; 2 China Animal Disease Control Center, Beijing, 100125 China

**Keywords:** African swine fever virus, wild boar lung cell line, multiomics, viral replication

## Abstract

African swine fever virus (ASFV) is a large double-stranded DNA virus that poses a significant threat to the global pig industry. Currently, our understanding of ASFV biology remains limited, as there is a lack of suitable cell lines to support its propagation and analysis. Here, we optimized a wild boar lung cell line to increase its susceptibility to ASFV, followed by temporally integrated transcriptomic and proteomic analyses of ASFV infection. Multiomics analysis revealed more than 17,000 genes and 5100 proteins, with 1594 differentially expressed genes (DEGs) and 923 differentially expressed proteins (DEPs) identified. Temporal dynamics revealed stage-specific host modulation: early-phase DEPs orchestrated metabolic reprogramming and transmembrane transport via the solute carrier superfamily (e.g., SLC25A, SLC30A, and SLC44A2), whereas the late infection phase featured concurrent upregulation of innate immune effectors (e.g., Mx1, OAS1, ISG15, and TRIM21) and suppression of apoptosis inhibitors (e.g., TMEM192, PDCD4, and DPP9), suggesting synchronized antiviral activation and apoptotic regulation. Unexpectedly, siRNA-mediated knockdown of key DEGs or DEPs involved in antiviral immunity, interferon signalling, DNA repair, genome stability, immune regulation, the inflammatory response and vesicular transport did not significantly affect viral replication. Only knockdown of the genes *STX17* and *ZNF512* significantly impaired ASFV replication. This systematic investigation provides a comprehensive framework for further studies of ASFV–host interactions, identifies candidate host dependency/support factors and establishes critical groundwork for the development of targeted antiviral interventions and next-generation vaccine platforms.

**Supplementary Information:**

The online version contains supplementary material available at 10.1186/s13567-025-01629-2.

## Introduction

African swine fever (ASF) is a highly contagious and fatal disease caused by ASFV, with a mortality rate that can reach 100% [1]. This disease is among the most destructive afflictions affecting both domestic and wild pigs. It poses a significant threat to global swine production, with no effective vaccines or antivirals currently available [2, 3]. ASF was first reported in Kenya in 1921 and later spread to Europe and Asia [4, 5]. The first case of ASFV infection in China was reported in 2018, and the epidemic quickly spread nationwide, resulting in significant economic losses for the pig farming industry [6, 7]. The observation that alterations frequently occur in the ASFV genome, such as large deletions in the MGF family genes and recombination of genotypes I and II [8], have intensified the challenges in ASF prevention and control. Therefore, investigating how ASFV interacts with host cells to promote viral replication and pathogenicity is crucial for its prevention and control.

ASFV is a large double-stranded DNA virus and the sole member of the family *Asfarviridae* [9]. As an obligate intracellular parasite, ASFV needs to interact with host cells to antagonize host antiviral responses and hijack host metabolic pathways to facilitate infection and pathogenesis. During the viral invasion phase, ASFV manipulates actin-driven macropinocytosis, clathrin-mediated endocytosis, and efferocytosis for cellular entry, which requires the hijacking of a series of host proteins, including the tyrosine kinase receptor AXL and the phosphatidylserine receptors TIM-4 and CD1d [10–14]. Moreover, endosomes and the microtubule motor complex transport system are utilized for virion disassembly and transport by the host transmembrane protein TMEM239 and the LC8 light chain of cytoplasmic dynein [15, 16]. ASFV also enhances DNA synthesis and regulates the cell cycle to promote viral replication [17]. Moreover, ASFV has evolved multiple mechanisms to antagonize the host immune response. It inhibits interferon production by degrading cGAMP or interfering with STING translocation and STAT1 phosphorylation [18, 19]. A comprehensive understanding of the interactions between ASFV and host cells will facilitate the elucidation of the underlying mechanisms of ASFV infection and pathogenesis.

Omics is a widely employed, high-throughput research methodology for investigating the impact of viral infections on host organisms and includes transcriptomics, proteomics, ubiquitinomics and acetylomics. Previous studies have reported alterations in the transcriptional levels of key host factors following ASFV infection in porcine alveolar macrophages (PAMs), 3D4/21 cells, and Vero cells [20–23]. Proteomic studies of ASFV-infected susceptible and nonsusceptible cell lines, pig tissues, and extracellular vesicles from ASFV-positive sera have revealed differential regulation of host proteins involved in immune and defence responses [24–28]. Nevertheless, none of the previous studies have captured the dynamic changes in infected hosts from multiple aspects, which limits our understanding of the ongoing biological processes during ASFV infection. Dynamic multiomics profiling of ASFV-targeted host cells could enhance our understanding of the mechanisms by which host cells respond to ASFV infection across multiple levels, including the genome, epigenome, transcriptome, proteome and metabolome.

The porcine macrophage–monocyte lineage is the main cell type targeted by ASFV. However, primary macrophages are challenging to prepare and handle, prone to bacterial and viral contamination, exhibit low gene editing efficiency and suffer from poor reproducibility because of batch-to-batch variability [29–31], which significantly hinders ASFV research. Therefore, the development of transmissible cell lines to support ASFV replication has been a research focus. Studies have shown that Vero, MA104, PK1, IPKM, and WSL-R (wild boar lung-derived cells) can support ASFV proliferation under specific conditions [32–35]. However, in these cell lines, ASFV has low replication efficiency and is prone to undergoing subtle to large genome deletions [36]. Therefore, the development of porcine-derived cell lines capable of optimal ASFV propagation is crucial for advancing fundamental research and improving vaccine production.

In this study, we optimized the WSL-R cell line to create an in vitro model with stable propagation and high ASFV infection efficiency. Through the analysis of both multiomics data and experimental results, we characterized the dynamic changes in WSL-R4 cells during ASFV infection and evaluated the correlation between transcriptional levels and protein expression. Combining functional clustering of DEPs with the key nodes in the protein‒protein interaction networks, we found that a series of conventional antiviral factors, as well as DNA replication and RNA transcription factors, had no significant effect on ASFV replication, except for ZNF512 and Syntaxin 17.

## Materials and methods

### Cells, viruses, and antibodies

WSL-R cells and their subclonal line (WSL-R4) were cultured in RPMI 1640 medium (#61870044; Gibco, New York, NY, USA) supplemented with 10% foetal bovine serum (FBS) (16140071; Gibco, New York, NY, USA), 50 U/mL penicillin, and 50 μg/mL streptomycin at 37 °C in a humidified atmosphere containing 5% CO_2_. The genotype II ASFV strain CADC_HN09 (ASFV-HN09) (GenBank accession no: MZ614662.1) and ASFV-GFP used in this study have been described previously [12].

Rabbit anti-STX17 polyclonal antibody (pAb) (ab316119; Abcam, Cambridge, UK) was purchased from Abcam. Monoclonal antibodies against the ASFV pp62 and p30 proteins were prepared in our laboratory.

### Subcloning and directed screening of WSL-R cell lines

WSL-R cells were cultured in a 10 cm^2^ dish and subcloned using limiting dilution after 48~72 h.  The cell count was used to determine the dilution volume, resulting in a final concentration of 5~10 cells/mL in RPMI 1640 medium supplemented with 10% FBS. The cell suspension was evenly distributed across a 96-well plate at 100 µL/well and incubated at 37 °C with 5% CO_2_ for 10~15 days. Once the single-cell clones formed cell clusters, they were trypsinized and split into two separate 96-well plates. One plate was used for continued culture, whereas the other was subjected to ASFV susceptibility testing. When the cell density reached 80~90%, the cells were infected with ASFV-GFP at a multiplicity of infection (MOI) of 1.0 and cultured for an additional 48 h. Afterwards, the GFP fluorescence intensity and cell counts were evaluated using a fluorescence microscope. The susceptibility of different subclonal cell lines was assessed on the basis of fluorescence intensity and infection rate (GFP-positive cells/total cells). The ASFV-susceptible cell lines were then selected from the corresponding wells of another 96-well plate. This process was repeated four times for subcloning and targeted screening. The selected cells were expanded and cultured in RPMI 1640 medium supplemented with 10% FBS and named WSL-R4 cells.

### Viral infections

WSL-R4 cells were seeded in 10 cm^2^ dishes and infected with ASFV-HN09 or ASFV-GFP (MOI = 1.0). After being incubated for 1 h at 37 °C, the cells were washed three times with medium to remove unbound viral particles. The cells were subsequently cultured in RPMI 1640 medium supplemented with 2% FBS and collected at the indicated time points post infection for further analysis. All experiments involving live ASFV were performed in the Biosafety Level 3 Laboratory at China Agricultural University (licence number: 2022-ASFV-002).

### Transcriptomic analysis

Three biological replicates of WSL-R4 cells were cultured in 10 cm^2^ dishes and either left uninfected or infected with the ASFV strain HN09 at an MOI of 1.0. The samples were collected for total RNA extraction and RNA sequencing at 6, 12, or 24 h post-infection (hpi). Total RNA was isolated from cell samples using TRIzol (Thermo Fisher, 15596026) following the manufacturer’s instructions. An RNA library was constructed, and sequencing was performed using an Illumina NovaSeq 6000 sequencer (Illumina, San Diego, CA, USA) with services provided by Beijing Tiangen Biotech Co., Ltd. (Beijing, China). Clean reads were mapped to the reference porcine transcriptome (*Sus_scrofa* 11.1). DEGs were identified using DESeq2.

### Proteomic analysis

The protein extraction procedure was as follows: as mentioned above, the culture medium was removed, and the cells were washed three times with 10 mL of prechilled PBS. Mock- or ASFV-infected WSL-R4 cells were then scraped and collected into 1.5 mL tubes. The cells were lysed with protein lysis buffer (8 M urea, 1% SDS with protease inhibitor) and sonicated for 2 min (min) to further solubilize the proteins. The cell lysate was incubated on ice for 30 min and then centrifuged at 12 000 rpm for 15 min at 4 °C to remove cellular debris. The protein concentration was then measured using a BCA kit according to the manufacturer's instructions.

The procedures for protein reductive alkylation and TMT labelling are described below. Briefly, 100 µg of protein was treated with triethylammonium bicarbonate buffer (TEAB) to achieve a final concentration of 100 mM. Then, Tris (2-carboxyethyl) phosphine (TCEP) was added to reach a final concentration of 10 mM, and the mixture was incubated for 60 min at 37 °C. Next, 40 mM iodoacetamide was added to achieve the final concentration, and the reaction was conducted in the dark for 40 min at room temperature (RT). Ice-cold acetone (V:V = 6:1) was added, and the reaction was incubated for 4 h at −20 °C. The supernatant was then removed by centrifugation at 10 000 × *g* for 20 min. The precipitate was dissolved in 100 µL of 100 mM TEAB and digested overnight at 37 °C with trypsin (M:M = 1:50). Finally, TMT was added to label the proteins for 2 h at RT, followed by treatment with hydroxylamine for 30 min.

### Data processing and analysis for proteomics

The raw files were analysed using Proteome Discoverer (PD) 2.2 software (Thermo Fisher Scientific, USA). The files were recalibrated using the *Sus scrofa* SwissProt database (TaxID: 9823). Spectra were selected with default settings, and database searches were conducted with the SequestHT node in PD. To enhance the quality of the analysis and reduce the false-positive rate, PD 2.2 software was used to apply additional filtering to the results. Peptide Spectrum Matches (PSMs) with a confidence level of 99% or higher were considered plausible, and proteins containing at least one UNIQUE peptide were classified as credible. Only credible spectral peptides and proteins were retained, and false discovery rate (FDR) validation was performed to remove peptides and proteins whose FDRs were greater than 1%. The results were exported to Excel files for further analysis. Log_2_ fold changes were calculated by applying a log_2_ transformation to the ratio of the mean of treated samples versus control samples. Statistical significance was determined using an unpaired, two-sided Student’s *t* test. *P* values were adjusted using Benjamini–Hochberg FDR correction. Adjusted *p* values (q values) less than 0.05 were considered significant.

Gene Ontology (GO) analysis of the DEPs was performed using the Ensembl Biomart database. The top 30 enriched pathways in each group are presented as bubble diagrams, with DEPs from these pathways displayed in a heatmap. Protein‒protein interaction (PPI) networks were analysed using the STRING database. Terms were considered valid if they met the following criteria: *p* < 0.05, |normalized enrichment score (NES)|> 1, and false discovery rate (FDR) < 0.25.

### Quantitative real-time PCR

To assess the interference efficiency of the siRNAs, WSL-R4 cells were transfected with 60 pmol of siRNA and incubated for 36 h. Total RNA was extracted from cultured cells using TRIzol reagent (Magen, MD020) following the manufacturer’s instructions. cDNA was synthesized from 1 μg of total RNA using the Hifair® AdvanceFast 1st Strand cDNA Synthesis Kit (Yeasen, 11149ES60) according to the manufacturer’s protocol. Quantitative PCR (qPCR) was performed in triplicate on a Bio-Rad CFX Maestro system (Bio-Rad, USA) with ChamQ Universal SYBR qPCR Master Mix (Vazyme, Q712-02-AA). The reaction conditions were 50 °C for 2 min and 95 °C for 5 min, followed by 40 cycles of 95 °C for 15 s (s), 56 °C for 15 s and 72 °C for 15 s. Cellular β-actin served as the internal control to normalize the cDNA levels. The 2^−ΔΔCt^ method was employed to calculate the relative abundance of target gene mRNA. The sequences of the primers used for qPCR are provided in Additional file 1.

### RNA interference (RNAi) assay

Small interfering RNAs (siRNAs) were designed to target different coding regions of each gene for RNA interference. The siRNA sequences are provided in Additional file 2. siRNAs were transfected using Lipofectamine® RNAiMAX (Thermo, 3,778,150) according to the manufacturer’s instructions. The knockdown effect was assessed by qPCR at 24 h post-transfection (hpt). The qPCR primers are shown in Additional file 1. Cellular β-actin was used as the reference gene. For the transfection/infection assay, WSL-R4 cells were infected with ASFV-HN09 or ASFV-GFP (MOI = 1.0) at 24 hpt, and total virus was collected at 36 hpi. The samples were titrated on WSL-R4 cells using the standard TCID_50_ assay.

### western blotting

Cells were washed twice with precooled PBS and lysed on ice using RIPA lysis buffer (Beyotime, #P0013C) supplemented with a protease inhibitor cocktail. Protein content was quantified using the Pierce™ BCA Protein Assay Kit (Thermo Fisher, #23225), followed by western blot analysis. Protein samples were separated by 10% SDS‒PAGE electrophoresis and transferred to PVDF membranes. The membranes were blocked for 1.5 h at room temperature with PBST (137 mM NaCl, 10 mM Na_2_HPO_4_, 1.8 mM H_2_PO_4_, 2.7 mM KCl (pH 7.4), and 0.1% Tween 20) supplemented with 5% skim milk powder and then incubated with the appropriate primary antibodies for 1 h at RT or overnight at 4 °C. The membrane was subsequently washed three times with PBST and incubated with a horseradish peroxidase (HRP)-conjugated secondary antibody for 1 h at RT. After three washes, the membrane was developed with Pierce ECL Western blotting Substrate (Thermo Fisher, #32209) and detected using a ChemiDoc MP Imaging System (Bio-Rad, USA).

### Statistical analysis

GraphPad Prism version 7.0 was used to perform the statistical analyses. Asterisks indicate statistical significance: *p < 0.05, **p < 0.01 and ***p < 0.001; N.S., not significant. The error bars indicate the ± standard deviation (SD).

## Results

### Development of a WSL-R4 cell line with high ASFV susceptibility via subclone-directed screening

**Figure 1:**
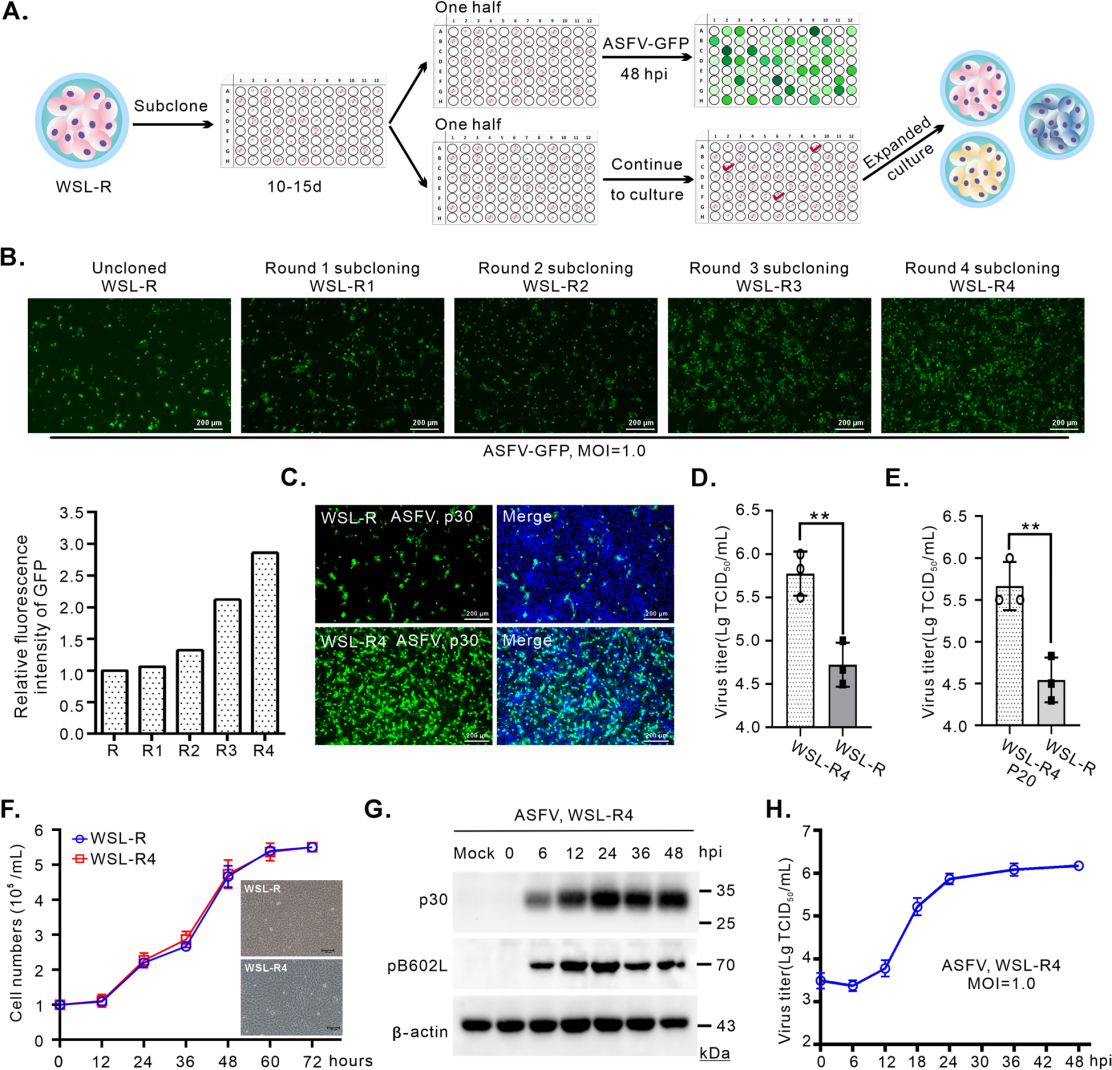
**Directed screening of WSL-R cell subclones for high susceptibility to ASFV**. **A** Workflow for screening WSL-R cells with ASFV-GFP. **B** Subcloned WSL-R cell lines were infected with ASFV-GFP (MOI = 1.0) for 48 h, and GFP signals were observed by fluorescence microscopy. Scale bar = 200 μm. The fluorescence intensity of each cell line was quantified using ImageJ. **C** WSL-R and subcloned cell lines were infected with ASFV-HN09 (MOI = 1.0) for 48 h, and the ASFV infection efficiency was assessed using a monoclonal antibody against the ASFV p30 protein. p30: green; DAPI: blue; scale bar = 200 μm. **D** Viral titres of parental WSL-R cells and the subcloned WSL-R4 cell line infected with ASFV-HN09. **E** WSL-R4 cells were infected with ASFV-HN09 after 20 consecutive passages, after which viral titres were measured. **F** Number of monoclonal parental WSL-R cells and the subcloned WSL-R4 cell line at different time points. **G** Western blot analysis of ASFV p30 and pB602L proteins in WSL-R4 cells at 0, 6, 12, 24, 36, and 48 h post-infection (hpi) with ASFV-HN09. **H** In vitro growth kinetics of ASFV-HN09 in WSL-R4 cells.

Currently, cell lines that support efficient ASFV infection and sustained passage are lacking. ASFV has been reported to infect WSL-R cells [35, 37] but with poor infection efficiency and low viral titres. To address this, we performed limited dilution for WSL-R single-cell cloning and assessed the ASFV susceptibility of different clones with a GFP-labelled wild-type ASFV strain HN09 derivative (ASFV-GFP) at loci encoding MGF360-18R under the control of the p72 promoter (Figure 1A). After single-cell clones were infected with ASFV-GFP at an MOI of 1.0, the infection efficiency of WSL-R4 significantly increased after four rounds of targeted selection (Figures 1A, B). We also evaluated the infection efficiency of the wild-type (WT) strain ASFV on WSL-R and WSL-R4 cells via indirect immunofluorescence and TCID_50_ assays. The results indicated that WSL-R4 cells were more susceptible than WSL-R cells, where a tenfold increase in viral titre and a TCID_50_ value of 10^6^/mL were observed (Figures 1C, D). This high susceptibility is stable, as significantly elevated viral titres were observed after 20 consecutive ASFV passages of WSL-R4 cells (Figure 1E). Next, the growth characteristics of WSL-R4 cells were assessed by comparing the numbers of nonclonal WSL-R and WSL-R4 cells at various time points (Figure 1F). The results reveale consistent growth rates for both cell types. Thus, the WSL-R4 clone exhibited stable properties and was selected for further study.

To systematically evaluate ASFV replication, WSL-R4 cells were infected with the ASFV strain HN09 at an MOI of 1.0, and the dynamic changes in viral proteins and viral growth at different time points were detected. As expected, the early protein p30 and mid-late protein pB602L of ASFV were expressed as early as 6 hpi and peaked after 24 hpi (Figure 1G). Similarly, the virus titre increased prior to 12 hpi, noticeably increased at 18 hpi, and subsequently plateaued after 24 hpi (Figure 1H). These results demonstrate that ASFV efficiently infects WSL-R4 cells and that a higher viral titre is achieved following replication. This observation also provides an important basis for determining future sampling time points for multiomics analysis.

### ASFV alters the transcriptome profile in the WSL-R4 cell line

**Figure 2:**
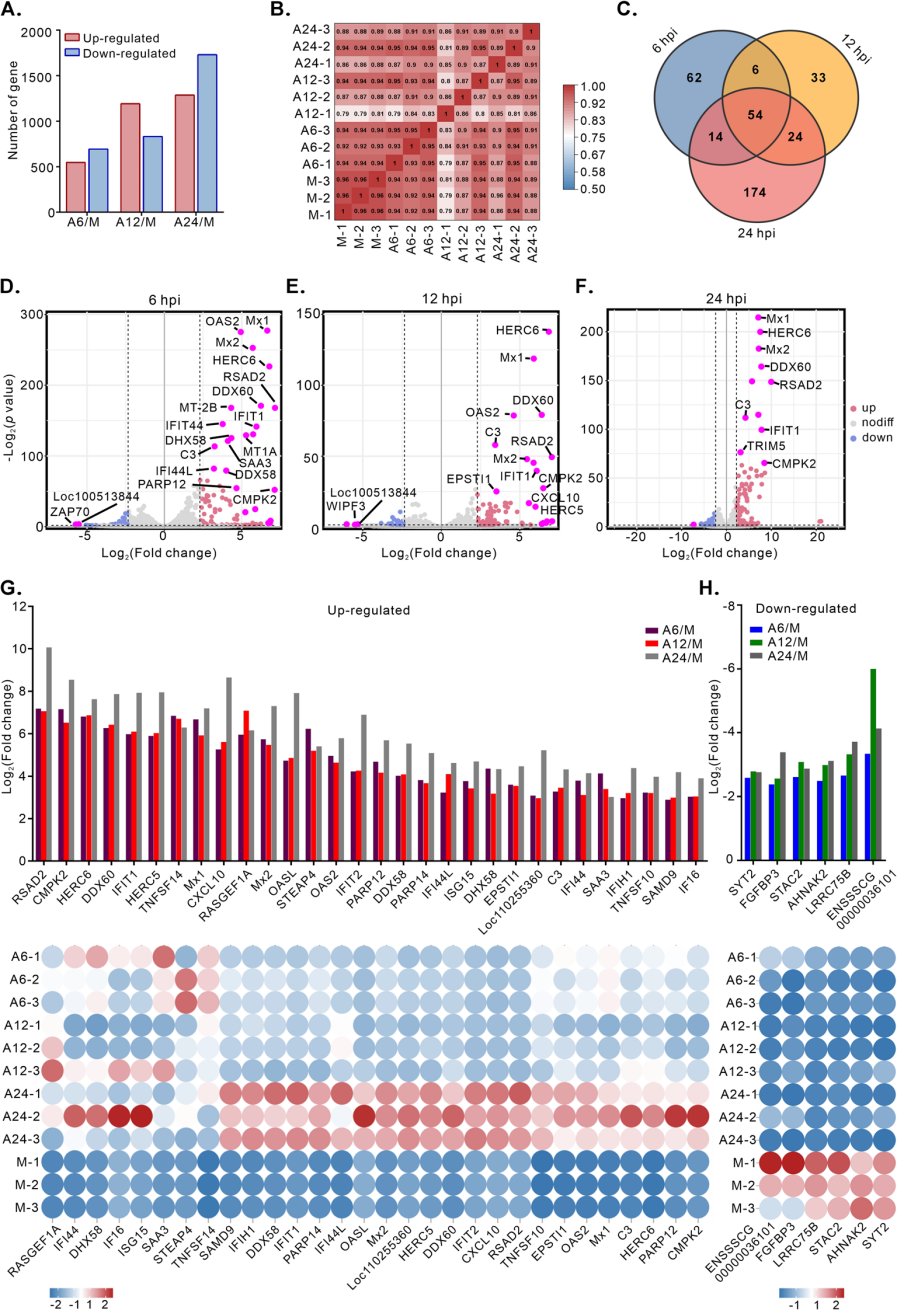
**Altered transcriptome profile in the WSL-R4 cell line at various time points post-ASFV infection.** WSL-R4 cells were infected with the ASFV strain HN09 at an MOI of 1.0 and harvested for transcriptome analysis at 6, 12, and 24 hpi. **A** Quantification of the number of DEGs. Fc ≥ 2 or Fc ≤ 0.5, *p* < 0.05. **B** Pearson correlation (R^2^) heatmap analysis of three biological replicates. **C** Venn diagram analysis of DEGs. Fc ≥ 5 or Fc ≤ 0.2, *p* < 0.05. **D**–**F** Volcano plots showing the fold changes and p values of the DEGs at 6, 12, and 24 hpi. Blue: downregulated genes, red: upregulated genes, grey: no significant difference, pink: selected genes of interest. **G**–**H** Heatmap representation of upregulated or downregulated genes. Fc ≥ 5, *p* < 0.05.

To characterize the cellular response of WSL-R4 cells to ASFV infection, we performed transcriptomic analysis at 6, 12, and 24 hpi with high-throughput RNA sequencing (RNA-seq). More than 17,000 genes were identified in the transcriptome. Genes whose expression significantly changed after ASFV infection (MOI = 1.0) were identified, including 1241 genes (547 upregulated and 694 downregulated) at 6 hpi, 2024 genes (1192 upregulated and 832 downregulated) at 12 hpi, and 2,917 genes (1287 upregulated and 1630 downregulated) at 24 hpi (Figure 2A; see Additional file 6). The Pearson correlation coefficient was not less than 0.8 within the three independent biological replicates at each time point, indicating a strong correlation and reproducibility (Figure 2B). To narrow down the results and identify the common DEGs, Venn diagrams were constructed to show the overlapping DEGs across infection stages, using a difference threshold of ≥ 5 (Figure 2C). Volcano plots of the DEGs revealed significantly upregulated genes with high variation, including Mx1, Mx2, OAS2, RSAD2, IFIT1, and DDX60 (Figures 2D–F). Most of these significantly upregulated genes were linked to host innate immunity. Notably, the expression of *RSAD2*, an IFN-stimulated gene, was upregulated more than one thousand-fold upon viral infection. It plays a key role in inhibiting the replication of various DNA and RNA viruses, including HCMV, HCV, WNV, dengue virus, Sindbis virus, and influenza A virus [38–41]. A total of 48 DEGs (only the first 30 are shown) were highly upregulated (*p* < 0.05, Fc ≥ 5) throughout the course of the infection (Figure 2G). Similarly, the significantly downregulated genes included SYT2, FGFBP3, STAC2, AHNAK2, LRRC75B, and a currently uncharacterized gene (ENSSSCG00000036101) (Figure 2H). AHNAK2 may regulate the PI3K–AKT pathway [42]. Overall, the transcriptomic data reveale that host cells activate the expression of multiple interferon-stimulated genes during viral infection.

To validate the differential transcription of ASFV-induced genes identified in the above sequencing data, we quantified the expression levels of eight previously predicted DEGs in mock- and ASFV-infected cells using quantitative real-time PCR. The transcriptional changes observed for these DEGs were consistent with those detected in the sequencing data (Additional files 4A–H), thereby confirming the robustness of our experimental design and bioinformatics analysis.

### ASFV induces altered proteomic profiles in the WSL-R4 cell line

**Figure 3:**
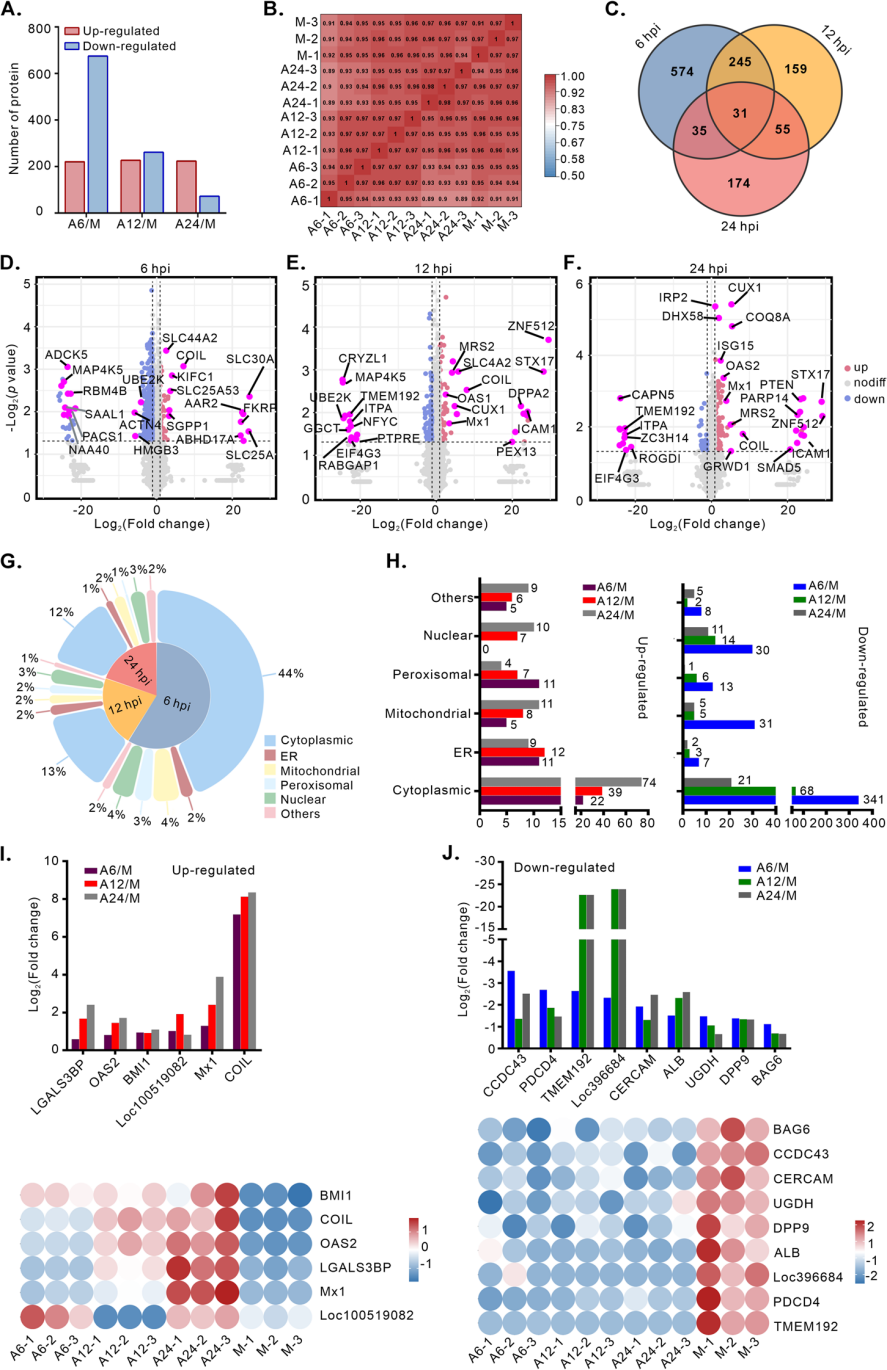
**Altered proteome profile in the WSL-R4 cell line at various time points post-ASFV infection**. WSL-R4 cells were infected with the ASFV strain HN09 at an MOI of 1.0 and harvested for proteome analysis at 6, 12, and 24 hpi. **A** Quantification of the number of DEPs. Fc ≥ 1.5 or Fc ≤ 0.67 and *p* < 0.05. **B** Pearson correlation (R^2^) heatmap analysis of three biological replicates. **C** Venn diagram analysis of the DEPs. Fc ≥ 1.5 or Fc ≤ 0.67 and *p* < 0.05. **D**–**F** Volcano plots showing the fold changes and p values of the DEPs at 6, 12, and 24 hpi. Blue: downregulated, red: upregulated, grey: no significant difference, pink: selected proteins of interest. **G**, **H** Subcellular localization of DEPs whose expression was upregulated or downregulated by 1.5-fold. **I**, **J** Heatmap representation of upregulated and downregulated proteins. Fc ≥ 1.5 or Fc ≤ 0.67 and *p* < 0.05.

To further understand the effect of ASFV infection on the cells, we also performed proteomic analysis at 6, 12, and 24 hpi. Among the 5100 quantified proteins, the number of proteins whose expression changed ≥ 1.5-fold decreased over time: 896 at 6 h, 489 at 12 h, and 297 at 24 h, primarily because of a significant reduction in the number of downregulated proteins (Figure 3A; Additional file 5). The Pearson correlation coefficient of 0.91 between biological replicates indicated high reproducibility (Figure 3B). Venn diagrams revealed 31 proteins that were significantly differentially expressed across all the stages of ASFV infection (Figure 3C). Volcano plots were used to display the DEPs at each time point (Figures 3D–F) and Gene Ontology annotation was used to classify proteins by subcellular localization (Figure 3G). Subcellular localization analysis revealed that approximately 69% of the total cellular proteins were located in the cytoplasm, surpassing those in the nucleus (10%), mitochondria (8%), peroxisomes (6%), and ER (6%). Fewer proteins were detected in the plasma membrane, Golgi apparatus, and lysosome. After ASFV infection, the changes in cytoplasmic protein expression shifted from downregulation to upregulation, likely because of the primary cytoplasmic replication of ASFV. Six proteins were significantly upregulated at various stages of ASFV infection (Figure 3I), with *COIL* showing the greatest increase (over 50-fold). *COIL*, a component of nuclear coiled bodies, is involved in modifying and assembling nucleoplasmic snRNPs. Additionally, the expression of Mx1/OAS2 was significantly upregulated, which is consistent with the transcriptomic data and confirms that ASFV infection activates the host antiviral response. In contrast, nine proteins, including TMEM192, PDCD4, and DPP9, which are associated with apoptosis inhibition, were consistently downregulated (Figure 3J), suggesting that ASFV infection induces apoptosis.

**Figure 4:**
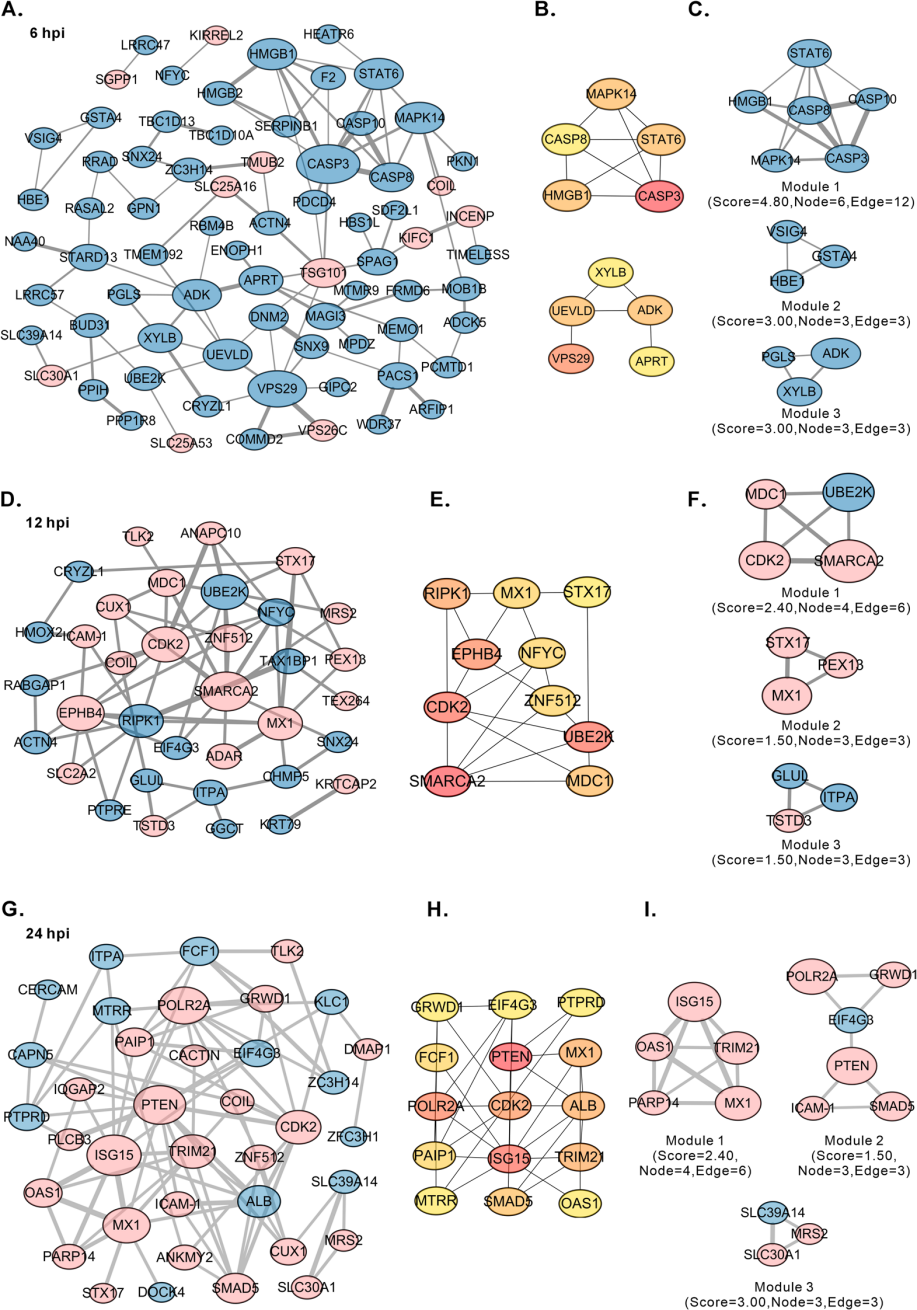
**Community discovery clustering network of DEGs in the integrated data from three time points**. **A**,** D**, and** G** Construction of a protein‒protein interaction (PPI) network of the DEPs. The blue nodes represent downregulated proteins, and the pink nodes represent upregulated proteins. The node size and line width are continuously mapped to the degree and combined score, respectively. **B**,** E**, and** H** Identification of Hub genes via the degree method in the CytoHubba plugin through the DEP PPI network. **C**,** F**, and** I** Selection of the most significant module by the MCODE plugin. The node size and line width are continuously mapped to the degree and combined score, respectively.

Viral infections involve PPIs, which can be represented as protein interaction networks (PINs), where proteins are nodes and their interactions are edges [43]. To examine the interactions between DEGs, we constructed PPI networks using Cytoscape. The 6-h PPI network contained 75 nodes and 96 edges (Figure 4A), with more than fivefold changes in protein expression in response to ASFV infection. Hub proteins were identified using Cytoscape’s CytoHubba. High-degree proteins were considered hub proteins with essential biological functions in the context of ASFV infection. The top 10 nodes with the greatest degree included CASP3 (score = 9), VPS29 (score = 8), ADK/UEVLD/STAT6/MAPK14/HMGB1 (score = 6), and APRT/XYLB/CASP8 (score = 5), all of which were downregulated in our proteomic analysis (Figure 4B). Cytoscape’s MCODE also revealed 3 modules, the most significant of which had a score of 4.80 and contained 6 nodes and 12 edges (Figure 4C). Similarly, compared with the 6-h upregulated nodes (14.7%), the 12-h PPI network had 35 nodes and 65 edges (Figure 4D), with 19 upregulated nodes (54.3%). The top 10 nodes with the highest degree at 12 h included SMARCA2 (score = 10), EPHB4/CDK2/UBE2K (score = 9), RIPK1 (score = 8), Mx1 (score = 7), and NFYC/MDC1/STX17/ZNF512 (score = 5) (Figure 4E). All the above proteins were upregulated except for UBE2K, RIPK1, and NFYC, whose expression was downregulated. The most significant module detected by MCODE consisted of 4 nodes and 6 edges, with a score of 2.40 (Figure 4F). In the 24-h PPI network, the greatest proportion of nodes were upregulated (24/37, 64.9%) (Figure 4G). The majority of the top 10 upregulated nodes were linked to innate immune responses, including ISG15 (score = 11), Mx1 (score = 8), TRIM21 (score = 8), and SMAD5 (score = 7) (Figure 4H). The most significant module detected by MCODE had a score of 5.00, with 5 nodes and 10 edges (Figure 4I). These results indicate that ASFV infection activates the innate immune response at 24 hpi. Furthermore, we validated the changes in the expression of STX17 during ASFV infection via western blot. The experimental findings are consistent with the proteomics data, demonstrating that STX17 is consistently upregulated throughout the ASFV infection process (Additional file 4I).

**Figure 5:**
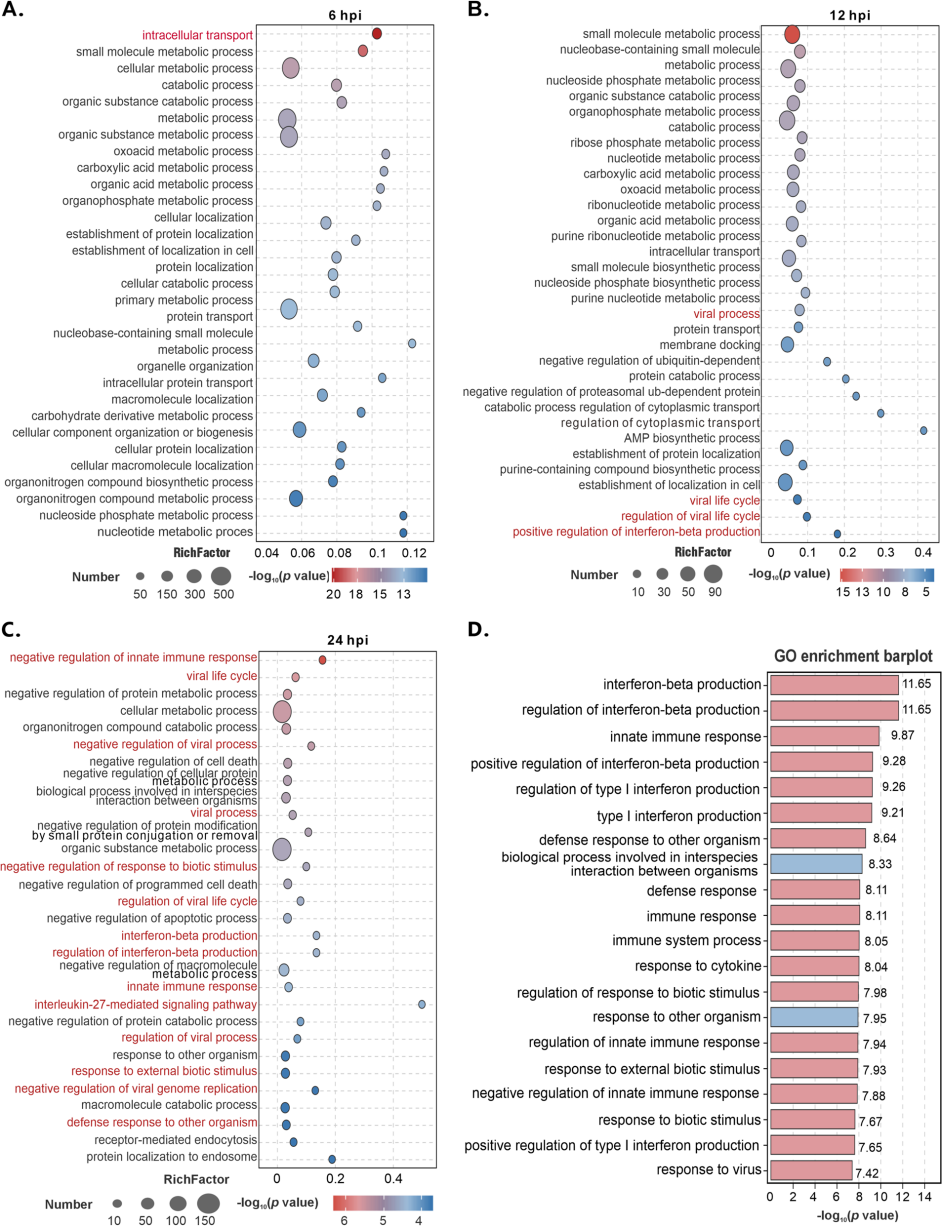
**GO analysis of DEPs at various time points after ASFV infection.** **A–C** The top 30 enriched terms from the GO analysis at 6, 12, and 24 hpi are presented. The size of the circled area is proportional to the number of DEPs. Antiviral and immune-related terms are highlighted in red. **D** The top 20 enriched terms from the GO analysis for the top 100 proteins with the highest degree in the PPI network. Antiviral and immune-related terms are highlighted in red, and irrelevant terms are shown in blue.

We continued the Gene Ontology (GO) enrichment analysis to explore the functional pathways related to the DEPs. In the GO analysis, we focused on the top 30 terms within the biological process (BP) subset. The results revealed distinct proteomic patterns at different stages of ASFV infection (Figures 5A–C, Additional file 6). At 6 hpi, the targeted pathways involved primarily host metabolic processes (Figure 5A), including those related to small molecules, organic compounds, oxygenated acids, and carboxylic acids. The quantified proteins were predominantly downregulated. Notably, the “intracellular transport process” term, which ranked first in enrichment, targeted a significant number of DEPs. Among these genes, six members of the solute carrier (SLC) superfamily (SLC30A1, SLC25A16, SLC25A53, SLC11A2, SLC44A2, and SLC30A7) were significantly upregulated, accounting for 11% of all proteins upregulated by at least two-fold. These results suggest that 6 h of ASFV infection can inhibit the host cell’s overall metabolic processes while promoting the transmembrane transport of substances. At 12 hpi, although the targeted pathways remained largely metabolic, virus-related and immune-related pathways were activated (Figure 5B). The pattern of DEPs in these pathways shifted from downregulated to predominantly upregulated. By 24 hpi, the proteomic pattern had shifted such that numerous antiviral immune responses were activated. The most enriched terms were related to the regulation of the viral life cycle and innate immune responses, with all the proteins involved being predominantly upregulated (Figure 5C). Similarly, GO enrichment analysis was conducted on the top 100 genes with the highest degree in the PPI network. Only two of the top twenty enriched terms were not related to antiviral or innate immunity (Figure 5D). Therefore, ASFV significantly activates the host antiviral immune response at the middle and late stages of infection.

### Comparison and correlation among the transcriptome and proteome

**Figure 6:**
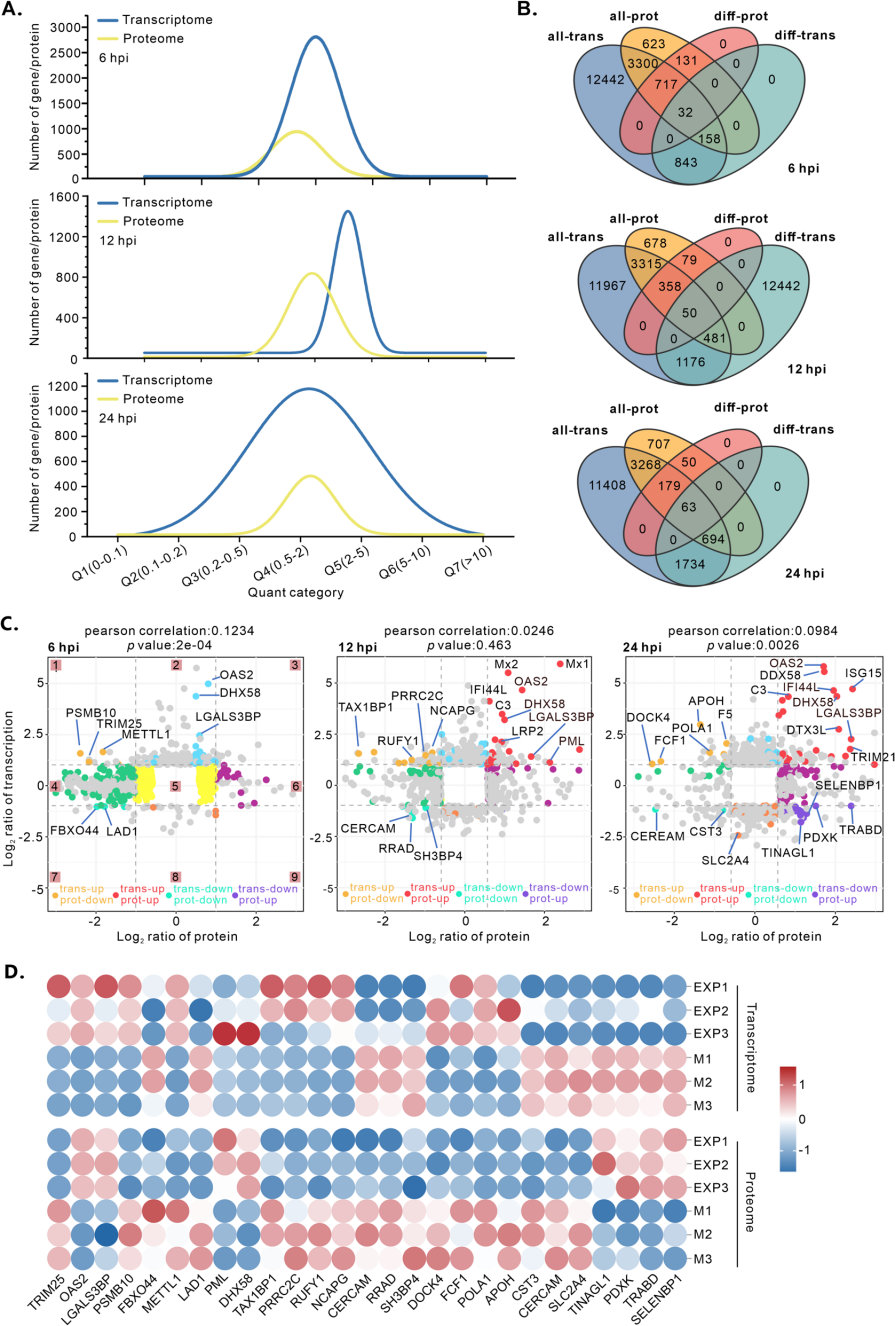
**Comparison and correlation among the transcriptome and proteome**. **A** Comparison of the distribution of the quantitative transcriptome and proteome. **B** Venn diagram analysis of the transcriptome and proteome. **C** Nine-quadrant plot of the correlation analysis between the transcriptome and proteome. **D** Protein expression levels of differentially expressed common genes from both the transcriptome and proteome, along with a heatmap representation.

To investigate the relationship between ASFV-induced changes in protein expression and gene expression, we compared the commonly expressed proteins and mRNAs across the three sampling time points. On the basis of the quantitative categories, the effects of different stages of ASFV infection on the numbers of DEGs and DEPs were examined and compared (Figure 6A). All genes and proteins (*p* < 0.05) were categorized by their fold change into seven quartiles (Q1–Q7): Q1 (0~0.1), Q2 (0.1~0.2), Q3 (0.2~0.5), Q4 (0.5~2), Q5 (2~5), Q6 (5~10), and Q7 (>10). The number of DEGs and DEPs varied significantly, and the fitted curve waveforms differed across stages, except at 24 h, when only a few correlations of DEPs and genes were observed (Figure 6B). At 6 hpi, 32 genes differed at both the transcriptional and translational levels. These genes were primarily involved in ribonucleoprotein complex biogenesis, RNA processing, and double-strand break repair via break-induced replication (Additional file 7). At 12 hpi, ASFV altered the expression of 50 genes at both the translational and transcriptional levels (Figure 6B). GO enrichment analysis revealed that the majority of enriched terms were related to physiological processes, such as defence responses to other organisms, interspecies interactions, and host defence responses to viral infection (Additional file 7). The 24-h phenotype was most distinct, as 26 of the top 30 enriched GO terms belonged to the biological process (BP) subset of 63 genes whose expression differed at both the transcriptional and translational levels. All of these genes were associated with host antiviral immunity (Additional file 7).

Additionally, we conducted a nine-quadrant analysis using the OmicShare platform to further explore the relationships between proteins and mRNAs at different stages of ASFV infection. Genes were highly enriched in the second quadrant, followed by the fourth quadrant (Figure 6C). Quadrants six, eight, and nine indicated that the expression abundance of proteins exceeded that of genes, possibly because of posttranscriptional or translational regulation. In quadrant five, proteins and RNAs were universally expressed without significant differences, whereas fewer proteins than their corresponding RNAs were expressed in quadrants one, two, and four. A portion of the DEGs and DEPs showed similar expression patterns in quadrants three and seven, particularly some interferon-stimulated genes (ISGs), suggesting that these genes were less influenced by posttranscriptional and translational regulation. Pearson correlation coefficients were calculated for 6 h (r = 0.1234), 12 h (r = 0.0246), and 24 h (r = 0.0984) (Figure 6C). These findings indicated a weak positive correlation between the transcriptome and proteome at different stages of ASFV infection. We analysed and compared the differential expression (*p* < 0.05) of 27 DEGs and DEPs across both datasets (Figure 6D). Notably, prominent antiviral proteins, such as TRIM25, OAS2, DHX58, LGALS3BP, and PML, were identified in both the transcriptome and proteome.

### Multiomics analysis has the potential to identify host restriction/support factors

**Figure 7:**
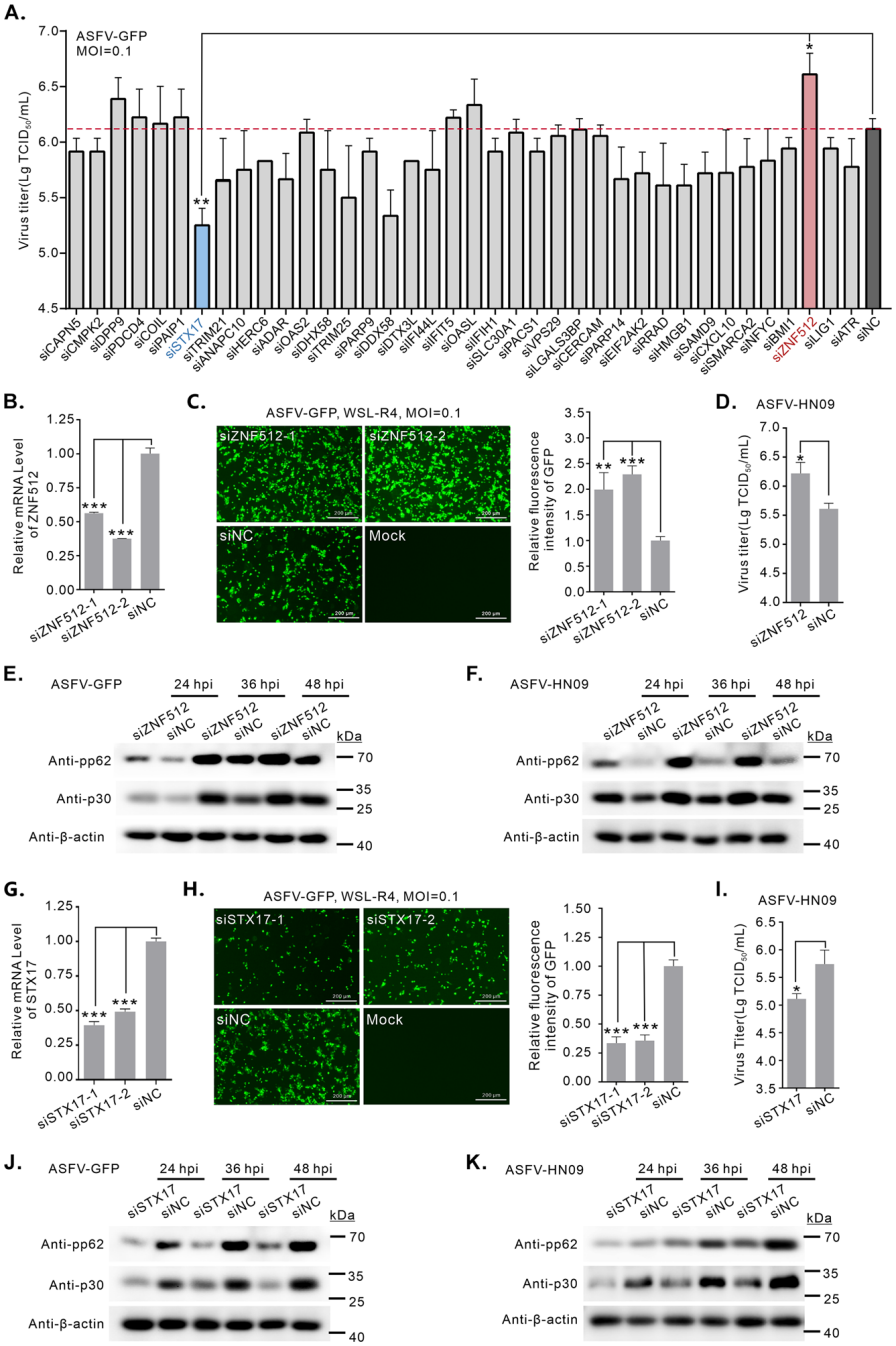
**siRNA screening of key host factors for ASFV replication on the basis of multiomics data**. **A** siRNA screening of key host factors for viral replication from the DEGs and DEPs. At 36 h post-transfection (hpt) with the indicated siRNAs, WSL-R4 cells were infected with ASFV-GFP at an MOI of 0.1. At 36 hpi, the whole culture was harvested for viral titration. **B** Analysis of the RNAi knockdown efficiency of the ZNF512 gene. WSL-R4 cells were transfected with siRNA targeting ZNF512, and the knockdown efficiency was assessed by qPCR at 36 hpt. The relative mRNA level of ZNF512 was normalized to that of β-actin and then compared to that of the siNC control. **C** Immunofluorescence analysis of the effect of ZNF512 knockdown on ASFV replication. The same as in (A), except that the cells were fixed to quantify the GFP signal intensity using ImageJ. **D** The same as (A), except that the cells were only transfected with siRNA targeting ZNF512 and infected with wild-type ASFV. **E**, **F** western blot analysis of the expression of viral proteins. At 36 hpt with siZNF512, the cells were infected with ASFV-GFP (**E**) or ASFV-HN09 (**F**) at an MOI of 0.1 and harvested at 24, 36, and 48 hpi for Western blot analysis with the indicated antibodies. **G** Analysis of the RNAi knockdown efficiency of the STX17 gene. The same as in (**B**), except that the siRNA target was changed to STX17. **H**,** I** Immunofluorescence analysis of the effect of STX17 knockdown on ASFV replication. The same as (**C**, **D**), except that the siRNA target was changed to STX17. **J**, **K** Western blotting analysis of the expression of viral proteins. The same as in (**E**, **F**), except that the siRNA target was changed to STX17.

Through functional clustering of DEGs and key PPI network nodes identified from multiomics data, we systematically designed siRNAs to screen for potential targets involved in antiviral immunity and interferon signalling, DNA repair and genome stability, protein ubiquitination and degradation, RNA metabolism and translation regulation, cell cycle regulation and apoptosis, immune modulation and inflammatory response, epigenetic regulation and chromatin remodelling, and vesicle transport (Table 1). The interference efficiency of each of the siRNAs was determined by RT‒qPCR, and the mRNA levels of the indicated genes were significantly reduced (Additional files 8A–H). The knockdown of most genes had a minimal effect on ASFV replication, with the exception of *ZNF512* (zinc finger protein 512) and *STX17* (Syntaxin 17) (Figure 7A). ZNF512, a zinc-finger protein, is involved in DNA binding, transcriptional regulation, and other gene expression-related cellular processes [44]. STX17, a soluble N-ethylmaleimide sensitive factor activator protein receptor (SNARE) protein, plays a role in autophagosome‒lysosome fusion and regulates the replication of various viruses [45].

**Table 1:** **Functional annotation for common DEGs**

Classification	Genes
Antiviral immunity and interferon signalling pathway	TRIM21, ADAR, OAS2, DHX58, TRIM25, DDX58, IFI44L, IFIT5, OASL, IFIH1, EIF2AK2, SAMD9, CXCL10
DNA repair and genome stability	PARP9, PARP14, LIG1, ATR, HMGB1
Protein ubiquitination and degradation	HERC6, TRIM25, DTX3L, ANAPC10
RNA metabolism and translation regulation	ADAR, PAIP1, EIF2AK2, COIL
Cell cycle regulation and apoptosis	PDCD4, CMPK2, CAPN5
Immunomodulation and inflammatory response	LGALS3BP, CERCAM, SLC30A1, PACS1, RRAD
Epigenetic and chromatin remodelling	SMARCA2, NFYC, BMI1, ZNF512
Vesicle transport	STX17, VPS29

The roles of ZNF512 and STX17 in ASFV replication were further investigated in a series of experiments. In the first assay, two additional siRNAs targeting ZNF512 and STX17 were designed and effectively silenced the gene expression, respectively (Figures 7B, G). Silencing ZNF512 expression significantly increased the infection efficiency of ASFV (Figure 7C). In contrast, STX17 knockdown strongly impaired ASFV replication (Figure 7H). In addition, the effects of silencing ZNF512 or STX17 on the WT-ASFV strain HN09 were tested in WSL-R4 cells. Consistent with that of ASFV-GFP, the replication of WT-ASFV was similarly influenced by these two host factors (Figures 7D, I). Moreover, the effects of silencing ZNF512 or STX17 on the viral protein expression of ASFV-GFP and WT-ASFV were detected. Compared with the control siRNA, ZNF512 knockdown significantly increased the expression of the ASFV matrix protein pp62 and structural protein p30 at various time points (Figures 7E, F). In contrast, knockdown of STX17 led to a reduction in viral protein expression (Figures 7J, K). The above results suggest that ZNF512 functions as a restriction factor for ASFV replication, whereas STX17 serves as a supportive factor.

## Discussion

ASFV causes substantial economic losses in the global swine industry, and effective control measures are lacking [2, 3, 46]. However, the biology and pathogenesis of ASFV remain incompletely characterized, hindering effective ASF prevention and control. In this study, we elucidated the dynamic regulatory network of ASFV using a newly established highly susceptible WSL-R4 cell model. Through time-committed multiomics analysis, we elucidated a biphasic regulatory pattern during ASFV infection that includes early-stage metabolic reprogramming followed by late-phase immune-apoptotic coordination. Integrated multiomics profiling systematically revealed critical host factors that modulate ASFV replication. These findings provide mechanistic insights into ASFV–host interactions and a theoretical framework for the development of rational antiviral strategies and innovative vaccine platforms.

ASFV is highly cytophilic, primarily targets monocytes and alveolar macrophages, and lacks passaged cell lines for ASFV research. Although many cell lines have been previously used for ASFV research, they have several drawbacks: (a) Virus adaptation leads to large genomic deletions [33, 47]. Vero cells were the first cell line used in vitro; however, as virus adaptation occurred, the ability of the virus to replicate in PAMs gradually diminished [47]. Similar effects were also observed in domesticated HEK-293T cells [32]. Genome comparison revealed that the missing MGF genes in ASFV-P121 were highly similar to those in BA71V [48]. (b) Descendant viral particles are defective. In 3D4/21 cells, although ASFV can enter cells post attachment, express early viral proteins and undergo genomic replication, it fails to produce infectious viral particles because of defective translation of late proteins [49]. In contrast, in WSL-R cells, the virus exhibited better replication stability, and the proteomic changes induced by ASFV infection were similar to those observed in primary cells [27] but with a relatively low infection efficiency. Here, we subcloned WSL-R cells and identified a monoclonal cell line, designated WSL-R4, which exhibits enhanced sensitivity to ASFV infection and a high viral titre (Figures 1A–D), thereby rendering WSL-R4 a valuable resource for ASFV research.

### Multiomics integration reveals the stepwise regulatory strategy of ASFV

Several transcriptomic and experimental studies have consistently revealed that ASFV infection triggers dynamic remodelling of antiviral and inflammatory transcriptional programs, despite strain- and tissue-dependent variations [20–22, 50–52]. Although a previous study demonstrated coordinated activation of inflammatory cytokine cascades and ISGs in ASFV-infected porcine tissues through synergistic host responses [52], contrasting evidence from virulent strains such as ASFV-Pig/HLJ/18 indicates potent immunosuppression through metabolic subversion to facilitate viral propagation [53]. This difference highlights the critical difference in the understanding of ASFV strain-specific modulation of host immunity. Our multistage RNA-seq analysis of the WSL-R4 infection model revealed the conserved upregulation of 48 core response genes across infection phases, including innate immune effectors (e.g., Mx1/2, HERC5/6, IFIT1/2, IF16, IFIT1, ISG15, and OASL) and chemokines (e.g., CXCL10 and TNFSF10/14). These findings corroborate earlier in vivo observations of the attenuated strain ASFV-HuB20 inducing rapid cytokine or chemokine activation in PAMs [20].

The most striking finding concerns the paradoxical activation of RNA virus surveillance mechanisms (e.g., IFIH1 and DDX58) by ASFV alongside canonical DNA-sensing pathways. This dual signalling engagement contrasts with the pathway specificity observed in herpesviruses [54, 55] but parallels recent findings in vaccinia virus [56, 57]. Such molecular crosstalk between DNA and RNA recognition systems may represent a type of evolutionary adaptation to counteract broad-spectrum host defences, potentially linked to the remarkable ecological persistence of ASFV. These observations suggest that the ASFV immunomodulatory strategy operates on a continuum between immune activation and suppression and is influenced by both viral determinants (strain virulence) and host microenvironments. The consistent activation of specific chemokines across infection models offers opportunities for targeted immunotherapies, while the identification of RNA sensor engagement reveals potential druggable targets within nucleic acid-sensing pathways.

Our temporal proteomic profiling not only supports but also significantly expands upon previous findings on ASFV-induced metabolic remodelling in PAMs [24]. While earlier studies established that ASFV promotes host metabolic activity in PAMs during infection [24], our temporally integrated analysis revealed critical stage-specific regulation: early-phase metabolic suppression (75% of proteins downregulated) transitions to late-phase immune activation. This biphasic pattern shows that ASFV strategically prioritizes resource acquisition (early) before deploying immune countermeasures (late). Notably, the temporal escalation of antiviral pathway enrichment in the GO analyses confirms the mechanistic basis of this phased strategy. During the early infection phase, metabolic transporter proteins (e.g., SLC family members; 6/54 upregulated proteins) were predominantly involved, aligning with the metabolic reprogramming pattern of the attenuated HuB20 strain, suggesting evolutionary optimization for nutrient acquisition. In contrast, the mid- to late stages showed progressive immune pathway activation. The sustained upregulation of COIL, the primary protein of the Cajal body (CB), further exemplifies this temporal control logic. Unlike HCMV’s immediate CB suppression via UL3/UL30 [58], ASFV progressively co-opts the CB-mediated RNA processing machinery, potentially synchronizing viral biosynthetic needs with host defence neutralization. The temporal segregation of these processes, namely, metabolic modulation preceding immune activation, differs from the synchronous pathway disruption seen in herpesviruses, potentially explaining the unique ecological persistence of ASFV.

Many DEGs were found in the transcriptome without corresponding changes in the proteome. Previous studies have shown that ASFV activates host innate immune effectors early in infection at the transcriptional level [20], which was also observed in this study. However, the protein levels of these effectors at 6 hpi were insignificant, indicating that ASFV likely utilized various posttranscriptional strategies to control cellular processes for its replication. Moreover, several gene transcripts exhibited opposite trends to those of their protein counterparts, suggesting a disconnect between the transcriptome and proteome in ASFV-infected cells. The expression patterns of different genes can be classified into two types: 1. Transcriptional upregulation with protein downregulation. The potential reasons could be categorized as follows: (a) Negative regulation of mRNA processing. (b) Reduced translation efficiency. (c) Posttranslational modifications. (d) Promotion of protein degradation. Genes in this category primarily regulate immune-inflammatory responses, RNA modification and posttranslational regulation. 2. Transcriptional downregulation with protein upregulation. The potential reasons can be classified as follows: (a) Enhanced mRNA availability through specific RNA-binding proteins, miRNAs, or other noncoding RNAs. (b) Increased protein stability and inhibition of protein degradation pathways prolong the protein half-life, allowing accumulation. (c) Increased protein synthesis rate. (d) Host negative feedback mechanisms, such as p53-mediated self-regulation.

### Multiomics integration offers insights for identifying host factors

A previous proteomic study revealed that the host metabolic pathway of ARG1–polyamine is important for virus replication [24]. Similarly, DEPs from ASFV-infected and uninfected pig serum samples revealed that C1QTNF3 knockdown inhibits ASFV replication by upregulating the proinflammatory factors IL-1β, IL-8, and IL-6 [25]. Omics analysis also revealed several known restriction factors, including APOBEC3 [59], the IFITM family [60], and the TRIM family [61]. Therefore, omics analysis has significant potential for investigating host factors for ASFV infection. However, knocking down antiviral genes (e.g., Mx1, OAS2 and TRIM25), except for DDX58, did not significantly affect ASFV replication. Surprisingly, knocking down DDX58, an antiviral factor, inhibited ASFV proliferation. Given that DDX58 is a putative RNA helicase involved in several cellular processes, including RNA binding and modification of RNA secondary structure, we hypothesized that DDX58 may be involved in ASFV genome transcription or other processes [62]. Additionally, interfering with certain enzymes critical for DNA replication did not affect ASFV. This may be due to the complexity of the ASFV genome, which encodes more than 150 proteins, many of which have immunosuppressive or replicative enzyme functions (e.g., pC962R, pG1211R, pE165R, and pP1192R), reducing host dependence. Interestingly, the viral titre of ASFV was significantly reduced when the expression of STX17, a vesicle transport-associated protein and membrane fusion protein from the SNARE family, was reduced [45]. The knockdown of other vesicle transport-related proteins also affected ASFV proliferation. These findings suggest that although ASFV encodes various proteins for efficient replication, it still relies on host vesicle transport for the release of virus progeny at the end of the replication cycle.

## Supplementary Information

 **Additional file 1. Primers for qPCR**. Primers for detecting siRNA knockdown efficiency.

 **Additional file 2. Sequences for RNA interference**. SiRNA sequences of candidate targets identified by multiomics analysis.

 **Additional file 3. DEGs in response to ASFV infection**. Temporal changes in the expression of all the genes were altered at 6, 12, and 24 hpi.

 **Additional file 4. Verification of the transcriptomic and proteomic data**. WSL-R4 cells were mock-infected or infected with the ASFV strain HN09 at an MOI of 1.0. At 36 hpi, the cells were harvested for qPCR assays with the indicated primers targeting different host genes. The relative mRNA levels of different genes were normalized to that of β-actin and then compared to those in the mock-infected group. The validated upregulated DEGs are (A) IFIT2, (B) Mx1, (C) RASGEF1A, (D) OASL, (E) IFIT2, (F) DDX58, and (G) RSAD2. (H) Validated downregulated DEG-AHNAK2. (I) Western blotting analysis of the expression of STX17.

 **Additional file 5. DEPs in response to ASFV infection.** Temporal changes in the expression of all the proteins were altered at 6, 12, and 24 hpi.

 **Additional file 6. GO BP enrichment of host DEPs with time series**. GO analysis of differentially expressed proteins at 6, 12, and 24 hpi, presenting data from the BP subset.

 **Additional file 7. GO enrichment of DEGs/DEPs at both the transcriptional and protein levels in a time series**. GO analyses were performed at 6, 12, and 24 hpi, targeting genes/proteins whose transcript and protein levels significantly differed.

 **Additional file 8. Analysis of the RNAi knockdown efficiency of the indicated genes**. WSL-R4 cells were transfected with the indicated siRNAs, and the knockdown efficiencies were assessed by qPCR at 36 hpt with the indicated primers. The relative mRNA levels of different genes were normalized to that of β-actin and then compared to that of the siNC control. The siRNAs are targeted to genes involved primarily in (A) antiviral immunity and interferon signalling, (B) Vesicle transport, (C) DNA repair and genome stability, (D) Protein ubiquitination and degradation, (E) Cell cycle regulation and apoptosis, (F) Immune regulation and the inflammatory response, (G) RNA metabolism and translation regulation, (H) Epigenetic and chromatin remodelling.
