# Supplementary figures and images for: Temporally integrated multiomics analysis elucidates intricate regulatory mechanisms of ASFV in a wild boar lung-derived clonal cell line
Source: Vet Res. 2025 Oct 14;56:193. doi: 10.1186/s13567-025-01629-2 (PMC12523111; doi:10.1186/s13567-025-01629-2)

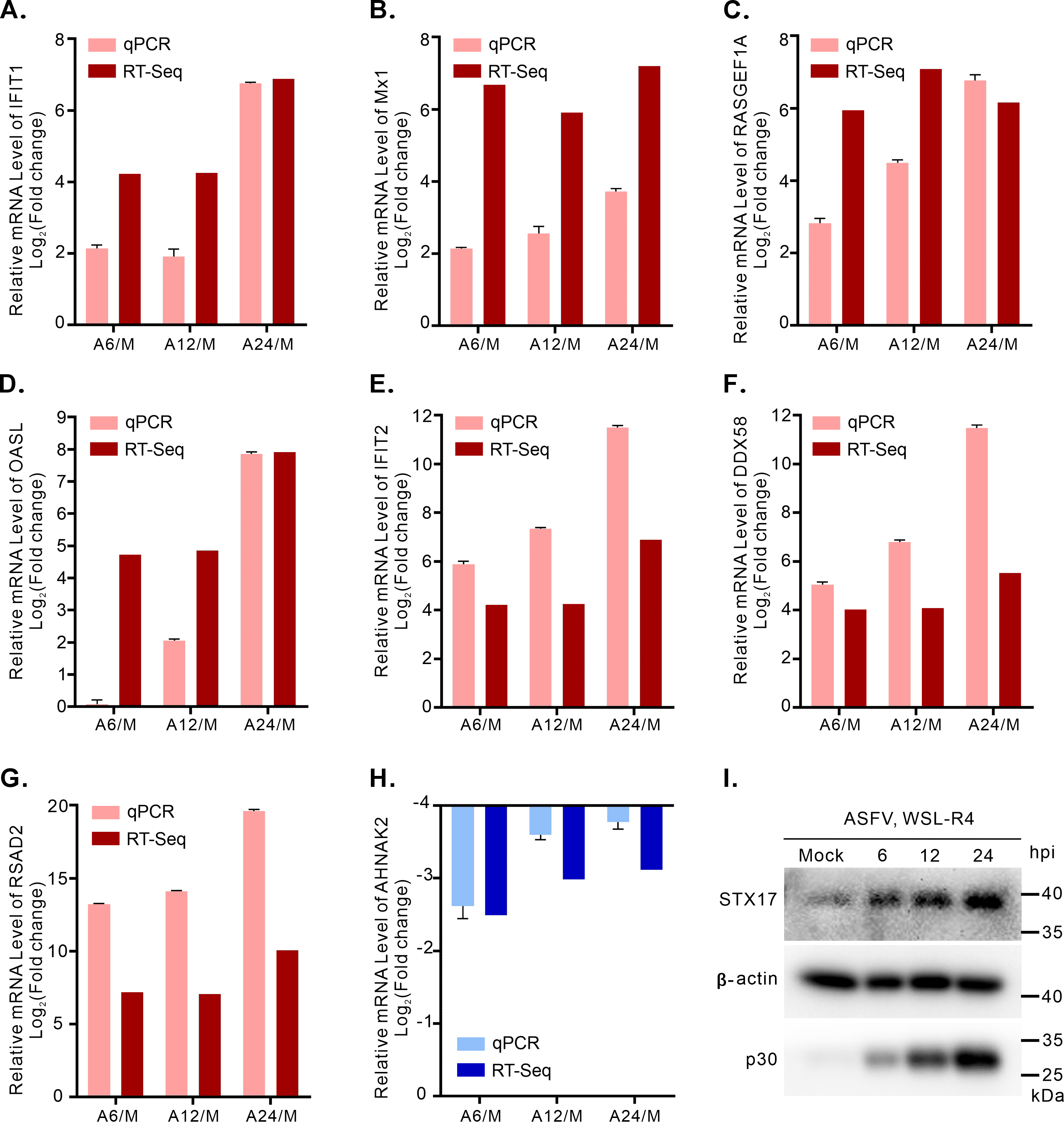

Supplement: Supplementary file 4 — Additional file 4. Verification of the transcriptomic and proteomic data. WSL-R4 cells were mock-infected or infected with the ASFV strain HN09 at an MOI of 1.0. At 36 hpi, the cells were harvested for qPCR assays with the indicated primers targeting different host genes. The relative mRNA levels of different genes were normalized to that of β-actin and then compared to those in the mock-infected group. The validated upregulated DEGs are (A) IFIT2, (B) Mx1, (C) RASGEF1A, (D) OASL, (E) IFIT2, (F) DDX58, and (G) RSAD2. (H) Validated downregulated DEG-AHNAK2. (I) Western blotting analysis of the expression of STX17. [file 13567_2025_1629_MOESM4_ESM.tif]

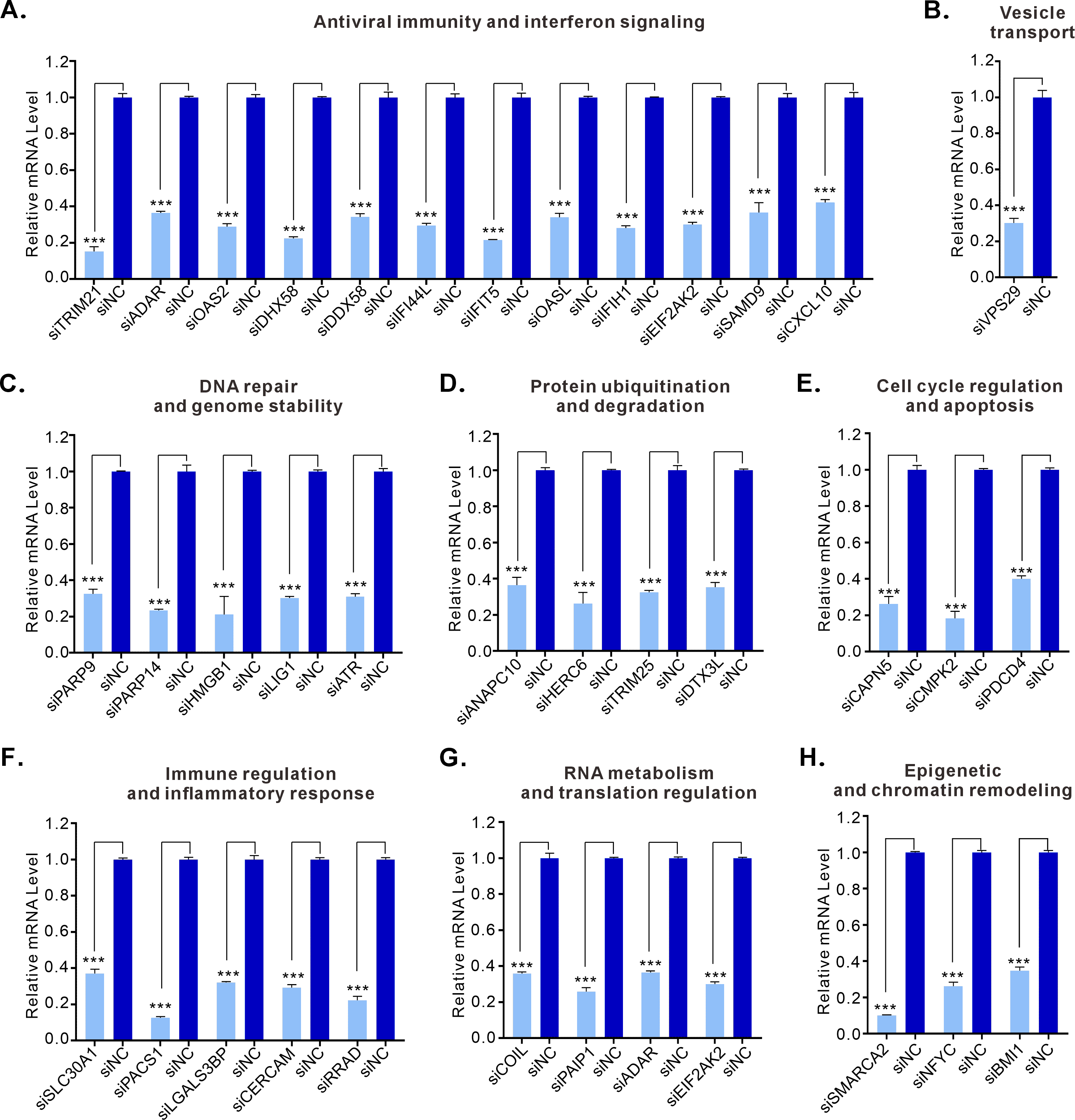

Supplement: Supplementary file 8 — Additional file 8. Analysis of the RNAi knockdown efficiency of the indicated genes. WSL-R4 cells were transfected with the indicated siRNAs, and the knockdown efficiencies were assessed by qPCR at 36 hpt with the indicated primers. The relative mRNA levels of different genes were normalized to that of β-actin and then compared to that of the siNC control. The siRNAs are targeted to genes involved primarily in (A) antiviral immunity and interferon signalling, (B) Vesicle transport, (C) DNA repair and genome stability, (D) Protein ubiquitination and degradation, (E) Cell cycle regulation and apoptosis, (F) Immune regulation and the inflammatory response, (G) RNA metabolism and translation regulation, (H) Epigenetic and chromatin remodelling. [file 13567_2025_1629_MOESM8_ESM.tif]
